# Prognostic factors associated with small for gestational age babies in a tertiary care hospital of Western Nepal: A cross‐sectional study

**DOI:** 10.1002/hsr2.250

**Published:** 2021-02-15

**Authors:** Nagendra Chaudhary, Shree Narayan Yadav, Suresh Kumar Kalra, Santosh Pathak, Binod Kumar Gupta, Sandeep Shrestha, Matthew Patel, Imran Satia, Steven Sadhra, Charlotte Emma Bolton, Om Prakash Kurmi

**Affiliations:** ^1^ Department of Pediatrics Universal College of Medical Sciences Bhairahawa Nepal; ^2^ Department of Pediatrics Chitwan Medical College Bharatpur Nepal; ^3^ The Royal College of Surgeons in Ireland Dublin Ireland; ^4^ Division of Respirology, Department of Medicine McMaster University Hamilton Ontario Canada; ^5^ Institute of Clinical Sciences College of Medical and Dental Sciences, University of Birmingham Birmingham UK; ^6^ NIHR Nottingham Biomedical Research Centre School of Medicine, University of Nottingham Nottingham UK; ^7^ Faculty of Health and Life Sciences University of Coventry Coventry UK

**Keywords:** appropriate for gestational age, household air pollution, prognostic factors, small for gestational age

## Abstract

**Background:**

Small for gestational age (SGA) is common among newborns in low‐income countries like Nepal and has higher immediate mortality and morbidities.

**Objectives:**

To study the prevalence and prognostic factors of SGA babies in Western Nepal.

**Methods:**

A cross‐sectional study (November 2016‐October 2017) was conducted in a tertiary care hospital in Western Nepal. Socio‐demographic, lifestyle factors including diet, and exposures including smoking and household air pollution in mothers who delivered newborns appropriate for gestational age (AGA), SGA and large for gestational age (LGA) were recorded. Logistic regression was carried out to find the odds ratio of prognostic factors after adjusting for potential confounders.

**Results:**

Out of 4000 delivered babies, 77% (n = 3078) were AGA, 20.3% (n = 813) were SGA and 2.7% (n = 109) were LGA. The proportion of female‐SGA was greater in comparison to male‐SGA (n = 427, 52.5% vs n = 386, 47.5%). SGA babies were born to mothers who had term, preterm, and postterm delivery in the following proportions 70.1%, 19.3%, and 10.6%, respectively. The average weight gain (mean ± SD) by mothers in AGA pregnancies was 10.3 ± 2.4 kg, whereas in SGA were 9.3 ± 2.4 kg. In addition to low socioeconomic status (OR 1.9, 95% CI 1.1, 3.2), other prognostic factors associated with SGA were lifestyle factors such as low maternal sleep duration (OR 5.1, CI 3.6, 7.4) and monthly or less frequent meat intake (OR 5.0, CI 3.2, 7.8). Besides smoking (OR 8.8, CI 2.1, 36.3), the other major environmental factor associated with SGA was exposure to household air pollution (OR 5.4, 4.1, 6.9) during pregnancy. Similarly, some of the adverse health conditions associated with a significantly higher risk of SGA were anemia, oligohydramnios, and gestational diabetes.

**Conclusions:**

SGA is common in Western Nepal and associated with several modifiable prognostic factors.

## What's already known?

SGA comprise a significant burden in low‐income countries with various modifiable risk factors.

## What this study adds?

In this observational study of newborn to Nepalese mother, one‐fifths of the babies were small for gestational age (SGA), with a higher proportion of female babies, and those born through caesarian section. Lifestyle factors such as lower intake of fruits and vegetables, smoking during pregnancy, and lower meat intake were associated with a higher risk for SGA. Exposure to household air pollution during pregnancy was associated with over 5‐fold increase risk of SGA. The findings from this study suggest the importance of addressing the issues with several modifiable risk factors that will reduce the proportion of SGA babies in Nepal.

## 1 INTRODUCTION

Small for gestational age (SGA) is the leading cause of low birth weight in low‐income countries (LICs).^1^ Globally, low birth weight and SGA at birth are associated with higher neonatal morbidity and mortality.^2^ In LICs, most children (14‐20 million annually) with low birth weight have intrauterine growth restriction (IUGR), is likely to be born preterm, a potential risk for SGA.^3^ In 2010, 32.4 million SGA infants were delivered worldwide, of which only 2.8 million were preterm infants.^4^ Globally, about 706 200 deaths in 2010 were attributed to SGA. More than half of global SGA births occurred in South Asia, with 44.5% of SGA prevalence in preterm births. In 2012, more than 80% of neonatal deaths in Sub‐Saharan Africa and South Asia were among small babies (65% attributable to preterm birth and 19% to term‐SGA). Approximately 10% of term infants in high‐income countries (HICs) are SGA, compared with 20% in LICs.^5^

SGA babies are more prone to hypoglycemia and hypothermia, which necessitates early recognition and immediate management. Despite optimal management of such babies, they are more likely to experience weak physical growth, poor neonatal neurodevelopmental outcomes, recurrent infections, and those surviving, in later life, are more likely to develop chronic diseases such as hypertension, obesity, hyperlipidemia, diabetes mellitus, and coronary heart disease.^6^

Nepal, a LIC in South Asia, has a neonatal mortality rate of 21 per 1000 live births, with over half associated with preterm birth‐related complications.^7^ The study on SGA and its determinants in Western Nepal is scarce. Therefore, our primary aim was to estimate the burden of SGA babies and the associated prognostic factors.

## 2 METHODS

### 2.1 Hospital setting and patient selection

We carried out a cross‐sectional study at Universal College of Medical Sciences, a 700 ‐bedded tertiary care hospital situated in province five of Western Nepal. The data acquisition process was carried out for about 10 months (November 28, 2016, to October 5, 2017). In this study, babies included delivered intramural live births from the labor room and postnatal wards or those admitted soon after delivery (within the first 24 hours from delivery) but delivered outside (extramural). Stillbirths were not included in this study. Verbal informed and written consents were taken from the parents of the enrolled babies in the study.

### 2.2 Sample size

The sample size calculation was based on an anticipated 22% prevalence of SGA babies^8^ to fall within 2% points of true proportion with a 99% confidence interval. The estimated sample size was 3723. We, therefore, included 4000 mother‐child pairs in our study.

### 2.3 Data collection tools

Data were collected by a medical doctor (pediatric resident) by filling in the information from the hospital medical records, interviewing mothers, and taking the required measurements where additional information was needed. The fellow clinical residents were trained in the data acquisition process for consistency of data collection.

An investigator‐administered questionnaire was used to collect information on the mother's details. Data were collected under three broad headings—lifestyle, environmental factors, and medical conditions of the mother. The investigator delivered questionnaire to collect information on socioeconomic status, diet, sleep duration during pregnancy, work during pregnancy, alcohol consumption, smoking status and passive smoking status, use of fuel (solid fuel or LPG) for domestic purposes, and passive smoking exposure. We collected current and past medical history on obstetric history, abortion or stillbirth, last menstrual period (LMP), gestational age, and expected delivery date (EDD). We measured the mother's hemoglobin level (less than <11 g/dl was considered anemia), weight, height, newborns' birth weight, length, head circumference, chest circumference, and umbilical cord stump length as part of standard medical care.

### 2.4 Study definitions and data collection technique

- Gestational age was estimated by the first day of the LMP and ultrasonography record (where available). In cases where LMP was unknown or in clinically discrepant cases, a clinical assessment confirmed it using New Ballard's scoring system. If the difference between LMP and scoring was more than 2 weeks, then the gestational age estimated by scoring was included.
- Gestational age of newborns were defined as: (a) extremely premature (born before completion of 28 weeks); (b) very early preterm (born between 28 weeks and <32 weeks); (c) moderate to late preterm (born between 32 and <37 weeks); (d) term (gestational age 37 0/7‐41 6/7 weeks); and (e) post term (gestational age 42 0/7 weeks or more). Term babies were further subclassified into early‐term (born between 37 and 38 weeks) and full‐term (born ≥39 weeks to <42 weeks).
- Birthweight was measured within 24 hours of birth using an electronic weighing machine (Goldtech, Precision Electronic Instruments Co., Delhi, India) to the nearest ±5 g.
- SGA, appropriate for gestational age (AGA), large for gestational age (LGA) classification: The classification of SGA, AGA, and LGA was defined as infants below the 10th centile, between 10 and 90th centile, and more than 90th centile of birth weight for gestational age, gender‐specific reference population respectively.^9^ We defined term‐SGA as a baby born small for gestational age at 37 or more completed weeks of gestation, and we classified preterm‐SGA as infants born small for gestational age at fewer than 37 weeks of gestation.
- Crown heel length was recorded to the nearest centimeter using infantometer.
- Head circumference (largest occipitofrontal diameter), chest circumference, and umbilical cord stump length were measured with a locally available measuring tape (fiberglass tape) in centimeters.

### 2.5 Statistical analysis

All the data were entered in the Microsoft excel 2007 and exported to STATA v16 for data cleaning, editing, and analysis. There were no missing data. Descriptive analysis included percentages and mean of different variables. For categorical variables, the *p*‐test was calculated using chi‐square, and for noncategorical data, the *t*‐test was used. Logistic regression was used to calculate the odds ratio of various parameters both for model 1 (adjusted for maternal age, maternal education, sex of the babies, maternal sleep duration, intake of different foods, solid fuel use for cooking, tobacco use, and environmental tobacco smoke) and model 2 (adjusted for model 1 + various existing maternal medical conditions).

### 2.6 Ethical approval

The study was approved by the Institutional Review Committee, Universal College of Medical Sciences, and Teaching Hospital (Ref. No. UCMS/IRC/010/17).

## 3 RESULTS

Out of 4000 mothers who delivered live babies, 52.2% were in the age group of 20‐24 years, 29.9% in the age group of 25‐29, and the remaining 17.9% were 30 or older. There was a significant increase in the caesarean section mode of delivery with the increase in the mothers' age group (20‐24 years, 25‐29, 30 or more were 33.7%, 42.4%, and 47.4%; respectively *p*‐trend <.001).

About 36.2% of the mothers in the age group 20‐24 years had full‐term deliveries compared to 41.3% in the age group 25‐29, and 42.4% in the age group 30 or more. The proportion of mother who has post term deliveries ranged from 8.9% (20‐24 years group) to 10.1% (30+ years group). The proportion of women with early‐term or moderate to late‐term deliveries was highest in the 20‐24 years group (52.6%) followed by 30+ years (49.6%) and lowest in 25‐29 years old group (46.3%). Women, who delivered SGA, were greatest in the age group 20‐24 years and gained the least weight during all three trimesters (Table 1).

**TABLE 1:** Comparison of AGA and SGA babies in relation to sex and maternal age

Characteristics	AGA	SGA	LGA
Maternal age (years)			
20–24	1594 (76.3)	450 (21.5)	44 (2.1)
25–29	939 (78.4)	221 (18.5)	37 (3.1)
30+	545 (76.2)	142 (19.9)	28 (3.9)
Weight gain [in kg (mean ± SD)]			
First trimester*	1.1 ± 0.8	1.1 ± 0.8	1.3 ± 0.9
Second trimester*	3.6 ± 0.8	3.3 ± 0.8	3.5 ± 0.8
Third trimester*	5.3 ± 0.8	4.9 ± 0.8	5.2 ± 0.8
Overall*	10.3 ± 2.4	9.3 ± 2.4	10.0 ± 2.6
Sex of the newborn			
Male*	1737 (79.2)	386 (17.6)	71 (3.2)
Female*	1341 (74.2)	427 (23.6)	38 (2.1)

Abbreviations: AGA, appropriate for gestational age; LGA, large for gestational age; SGA, small for gestational age.

* *P*‐trend for SGA, AGA and LGA <.001.

### 3.1 Characteristics of babies

The proportion of preterm, term, and postterm babies were 20.7% (n = 831), 70% (n = 2799), and 9.3% (n = 370) respectively. 76.9% (n = 3078) babies were AGA, 20.3% (n = 813) were SGA, and 2.7% (n = 109) were LGA. Among the SGA babies, 70.1% (n = 570) were term‐SGA, 19.3% (n = 157) were preterm‐SGA, and the remaining postterm SGA. Table 2 reports the comparative characteristics of SGA and AGA newborns. There were more females than male‐SGA babies (24.1% vs 18.2%), more were delivered through lower segment caesarean section (LSCS) compared to vaginal delivery (23.5% vs 19.2%), and had lower anthropometry indices (Table 2). The mean weights of SGA babies were about 800 g less compared to AGA babies.

**TABLE 2:** Baseline characteristics of newborn

Characteristics of newborn				
Characteristics	Small for gestational age (*N*, %)		Total (col. %)	*P*‐value (no vs yes)
	No (AGA)	Yes		
Gender				
Male	1737 (81.8)	386 (18.2)	2123 (54.6)	<.001
Female	1341 (75.8)	427 (24.1)	1768 (45.4)	
Mode of delivery				
NVD	1927 (80.8)	459 (19.2)	2386 (61.3)	.001
LSCS	1151 (76.5)	354 (23.5)	1505 (38.7)	
Anthropometry	*N* = 3187	*N* = 813	*N* = 4000	
Occipitofrontal circumference (mean ± SD in cm)	33.6 ± 1.8	30.8 ± 1.9	33.0 ± 2.1	<.001
Chest circumferences (mean ± SD in cm)	30.8 ± 2.2	27.2 ± 2.0	30.0 ± 2.6	<.001
Body length (mean ± SD in cm)	48.4 ± 3.1	44.2 ± 2.3	47.6 ± 3.5	<.001
Weight (mean ± SD in g)	2810.4 ± 507.7	2035.5 ± 438.8	2648.5 ± 586.0	<.001

Abbreviation: AGA, appropriate for gestational age; LSCS, lower segment cesarian section; NVD, normal vaginal delivery

### 3.2 Prognostic factors

Table 3 shows the socio‐demographic, lifestyle factors including diet, and exposures to cigarette smoke and household air pollution in mothers who delivered newborn with AGA, SGA, and LGA. Mothers with lower secondary education or less (compared to those with college or above), slept 5‐7 hours per day during pregnancy (compared to 10‐14 hours), monthly or less frequent intake (compared to daily intake) of fruits, fish, meat, and high carbohydrate snacks were associated with a higher risk of delivering SGA babies compared to AGA following adjustment for potential prognostic factors. Similarly, those mothers who smoked during the pregnancy, were exposed to passive smoking, or used solid fuel (exposure to HAP) to cook were independently associated with a higher risk of delivering SGA compared to AGA (Table 3). There was no significant difference directionally following the adjustment of various existing health conditions (model 2 in Table 3).

**TABLE 3:** Showing comparison of various prognostic factors with SGA babies

Characteristics	Classification according to gestational age			Model 1 OR (95%CI)	Model 2 OR (95%CI)	*p*‐trend for odds ratio
	AGA	SGA	LGA			
Sleeping duration (in h)						
10–14	1076 (88.1)	112 (9.2)	33 (2.7)	Ref	Ref	<.001
8–9	1591 (77.2)	418 (20.3)	53 (2.6)	2.7 (2.1‐3.6)	2.4 (1.8‐3.4)	
5–7	411 (57.3)	283 (39.5)	23 (3.2)	7.3 (5.4 9.9)	5.1 (3.6‐7.4)	
Education						
College or above	450 (79.8)	34 (6.0)	80 (14.2)	Ref	Ref	<.001
Secondary level	779 (91.0)	50 (5.8)	27 (3.1)	0.5 (0.3‐0.9)	0.4 (0.2‐0.8)	
Lower secondary or primary or no education	1839 (71.6)	729 (28.4)	‐	3.2 (2.0‐5.2)	1.9 (1.1‐3.2)	
Fruit intake frequency						
Daily	1927 (91.9)	88 (4.2)	82 (3.9)	Ref	Ref	<.001
Weekly	1002 (78.0)	260 (20.2)	22 (1.7)	5.2 (3.9‐6.9)	4.5 (3.2‐6.4)	
Monthly or less	149 (24.1)	465 (75.1)	5 (0.8)	67.2 (46.9‐96.5)	68.8 (44.4‐106.7)	
Fish intake frequency						
Weekly	2091 (86.2)	251 (10.3)	83 (3.4)	Ref	Ref	.001
Monthly or less	987 (62.7)	562 (35.7)	26 (1.6)	1.4 (1.1‐1.8)	1.7 (1.2‐2.4)	
Meat intake frequency						
Daily	811 (89.2)	61 (6.7)	37 (4.1)	Ref	Ref	<.001
Weekly	1716 (85.9)	222 (11.1)	60 (3.0)	1.70 (1.2–2.4)	1.5 (1.0‐2.3)	
Monthly or less	551 (50.4)	530 (48.5)	12 (1.1)	6.1 (4.2‐8.8)	5.0 (3.2‐7.8)	
Dairy product intake frequency						
Daily	1470 (79.0)	333 (17.9)	58 (3.1)	Ref	Ref	.628
Weekly	1485 (76.1)	418 (21.4)	48 (2.5)	0.9 (0.7‐1.1)	1.0 (0.8‐1.3)	
Monthly or less	123 (65.4)	62 (33.0)	3 (1.6)	1.3 (0.8‐2.1)	1.2 (0.7‐2.2)	
High carb snacks						
Daily	1748 (85.8)	215 (10.6)	73 (3.6)	Ref	Ref	<.001
Weekly	1221 (77.4)	323 (20.5)	33 (2.1)	2.1 (1.7‐2.6)	2.4 (1.8‐3.1)	
Monthly or less	108 (28.0)	275 (71.2)	3 (0.8)	24.8 (18.0‐34.1)	28.0 (19.1‐41.0)	
Smoking status						
Nonsmoker	3074 (77.4)	788 (19.8)	109 (2.7)	Ref	Ref	.003
Smoker	4 (13.8)	25 (86.2)	‐	24.7 (6.7‐91.5)	8.8 (2.1‐36.3)	
Exposure to passive smoking						
No	2814 (77.5)	717 (19.7)	101 (2.8)	Ref	Ref	.045
Yes	264 (71.7)	96 (26.1)	8 (2.2)	1.4 (1.0‐1.9)	1.5 (1.0‐2.2)	
Main fuel used for cooking						
LPG or electricity	2377 (85.7)	295 (10.6)	102 (3.7)	Ref	Ref	<.001
Solid fuel	701 (57.2)	518 (42.2)	7 (0.6)	5.5 (4.5‐6.8)	5.4 (4.1‐6.9)	

Abbreviations: AGA, appropriate for gestational age; LGA, large for gestational age; SGA, small for gestational age.

*Note*: Model 1: Adjusted for age group, sex of babies, maternal age, maternal sleep, education, high carb snack, solid fuel use, smoking, environmental tobacco smoking. Model 2: Model 1 + pregnancy‐induced hypertension, gestational diabetes, cardiovascular diseases, polyhydramnios, hypothyroid, and anemia.

Table 4 shows a comparison of maternal medical conditions that may be an independent predictor for SGA. Gestational diabetes (OR 2.5, 95% CI 1.5, 4.2), oligohydramnios (OR 5.0, 95% CI 3.8, 6.7), anemia (OR 19.6, 95% CI 14.6, 26.4), and presence of rash (OR 3.2, 95% CI 2.3, 4.6) were independent prognostic factors for SGA occurrence compared to AGA. Although a high proportion of mothers with cardiovascular disease, polyhydramnios, and hypothyroid had SGA babies compared to AGA, it was statistically not significant.

**TABLE 4:** Comparison of maternal medical conditions with SGA, AGA, and LGA

Maternal conditions	AGA	SGA	LGA	Model 1 OR (95% CI)	Model 2 OR (95% CI)	*P*‐value
Pregnancy‐induced hypertension	252 (8.2)	118 (14.5)	13 (11.9)	2.0 (1.6‐2.9)	0.9 (0.6‐1.3)	.290
Gestational diabetes	144 (4.7)	81 (10.0)	21 (19.3)	2.0 (1.4‐3.0)	**2.5 (1.5‐4.2)**	**.002**
Cardiovascular diseases	30 (1.0)	13 (1.6)	5 (4.6)	1.3 (0.5‐3.2)	1.7 (0.6‐4.6)	.105
Oligohydramnios	396 (12.9)	355 (43.7)	12 (11.0)	5.0 (4.0‐6.3)	**5.0 (3.8‐6.7)**	**<.001**
Polyhydramnios	52 (1.7)	33 (4.1)	7 (6.4)	2.0 (1.1‐3.5)	1.7 (0.7‐3.8)	.473
Hypothyroid	201 (6.5)	77 (9.5)	3 (2.7)	1.5 (1.0‐2.1)	1.3 (0.8‐2.0)	.188
Anemia	128 (4.2)	458 (56.3)	22 (20.2)	21.6 (16.3‐28.5)	**19.6 (14.6‐26.4)**	**<.001**

Abbreviations: AGA, appropriate for gestational age; LGA, large for gestational age; SGA, small for gestational age.

*Note*: SGA was dependent variable compared against AGA as a reference category. Model 1: Adjusted for age group, sex of babies, maternal age, maternal sleep, education, high carbohydrate snacks, solid fuel use, smoking, environmental tobacco smoking. Model 2: Model 1 + pregnancy‐induced hypertension, gestational diabetes, cardiovascular diseases, oligohydramnios, polyhydramnios, hypothyroid, and anemia

Gestational diabetes, oligohydramnios and anemia were independent prognostic factors for SGA occurence (denoted by bold values)

## 4 DISCUSSION

This study aimed to investigate the prevalence and prognostic factors for the development of SGA in Nepal. The prevalence of SGA in the present study was 20.3%. The occurrence of SGA was dependent on various modifiable prognostic factors such as lifestyle, environment, and health status of mothers.

The prevalence of SGA babies in low‐ and middle‐income countries (LMICs) is high, with two‐thirds of SGA babies born in Asia (17.4 million in South Asia). Lee et al. (2010) conducted a prevalence study in 138 LMICs and found that 32·4 million infants were born SGA, of whom 10·6 million infants were born at term and had low birth weight. Most SGA infants were born in India, Pakistan, Nigeria, and Bangladesh.^5^ Although the overall prevalence of SGA estimated was 27% of live births, Nepal's reported prevalence was higher (39.3%) than the present study (20.3%). Conversely, data from the WHO Multicountry Survey on Maternal and Newborn (2010‐2011)^10^ and a recent (2017‐2018) multisite study in 12 different hospitals^11^ reported a lower prevalence of SGA in Nepal (17.9% and 11.9%, respectively). The decreasing trends in the prevalence of SGA in Nepal could be due to advancement in Nepal's medical sector in the recent years with improvement in mothers' education, higher socioeconomic status, and a better understanding of the importance of antenatal visits during pregnancy.

In the present study, the proportion of babies with term‐SGA was 14.2% of live births, which were higher than East‐Asia (5.3%), North Africa (1.2%), and Southeast Asia (3.0%) but lower than in combined South Asia (41.5%).^5^ Our study found the proportions of term‐SGA more than the preterm‐SGA among all SGA babies (70.1% vs 19.3%). Similarly, Lee et al,^5^ in a large sample size of 18 million low‐birth‐weight babies, reported that 59% were term‐SGA, and 41% were preterm. This necessitates various antenatal steps, like maternal education and nutritional measures, to improve the newborn outcome. In contrast, a study conducted in China by Chen et al. (2010‐2012) showed the prevalence of preterm‐SGA higher than the term SGA.^12^

There are many different birth‐weight‐for‐gestation reference populations, used extensively to calculate the prevalence of SGA. Katz et al^13^ reported, depending upon the reference populations, the prevalence of SGA ranged from 10.5% to 72.5% in Nepal, and 12.0% to 78.4% in India. Thus, highlighting the importance of country‐specific curves to be considered in establishing the diagnosis of SGA. The proportion of SGA in male babies in our study was lower than females (17.6% vs 23.6%), as has also been reported by several studies,^11^, ^14^, ^15^, ^16^ unlike Muhihi et al,^17^ who reported a higher risk of SGA in male.

Various prognostic factors such as socioeconomic status, lifestyle factors, environmental factors, and the mother's medical conditions were assessed in this study. A study conducted in Pakistan^18^ reported that illiterate mothers had high chances of having an SGA baby (OR 2.1, 95% CI 1.2, 3.6), similar to our findings (OR 1.9, 95% CI 1.1, 3.2). Similarly, a study from Tanzania reported a decrease in SGA births with improved maternal education.^17^ The low chances of SGA in literate mothers could be due to them being better informed of maternal well‐being, and it impacts on the child. Therefore, improving educational measures and interventions may significantly reduce the SGA burden in LICs like Nepal.

Some studies have reported that women >35 years had high chances of delivering SGA babies. In contrast, others have found that advanced maternal age was either not a risk factor for SGA, or the effect of maternal age was no longer significant after adjustment for associated factors.^19^, ^20^ In the present study, the proportion of SGA babies was the highest in the age group 20‐24 years compared to 25‐29 years and 30+ years.

### 4.1 Lifestyle and environmental factors

Previous studies have demonstrated that cigarette smoking is causally associated with SGA and has a dose‐response relationship.^21^ In the present study, smoking during pregnancy was considered an independent risk factor with around 9‐fold higher risk; however, we had very few women who smoked and the wide confidence interval. A study reported that women who had stopped smoking by 15 weeks' gestation had an SGA rate equivalent to that of nonsmokers^22^; however, we do not have information on smoking cessation during the pregnancy. We also found that environmental tobacco smoking (passive smoking) was associated with a significantly higher risk of SGA (vs AGA), as reported by a study from India.^23^

In the present study, mothers who slept less (5‐7 hours or 8‐9 hours in contrast to 10‐14 hours) during the gestation period had over 5‐folds increased risk of having SGA babies. Similar findings have been reported by Abeysena et al^24^ In contrast, a recent study done by Morokuma et al^25^ reported no association between sleep duration on SGA occurrence necessitating the need for future longitudinal studies on maternal sleep and fetal outcome.

Previous studies have reported that exposure to smoke from solid fuel burning for cooking has increased risk for both LBW and SGA.^26^, ^27^ Our study's findings confirm the earlier results. Mothers exposed to smoke from solid fuels cooking were positively associated with higher SGA deliveries (AOR 5.4, 95% CI 4.1, 6.9), warranting the need for alternative cleaner fuels be used during cooking.

### 4.2 Dietary factors

The present study also aimed to study the effect of different dietary factors on SGA. A study conducted in India by Rao et al^28^ showed that fruit consumption at 28 weeks was associated with higher birth weight. A study from New Zealand^29^ reported that a low intake of fruit around the time of conception was associated with a tendency to increase SGA prevalence (AOR 1.49, 95% CI 1.0, 2.24). A similar finding was observed in the present study, mothers who consumed fruits once a month or once a week had higher chances of having an SGA baby than mothers who consumed fruits daily.

A Danish national birth cohort study showed that daily milk consumption was inversely associated with SGA.^30^ A study from the United States reported that milk consumption resulted in small increases in birth weight.^31^ However, our study reported a higher risk among those who consumed dairy products once a month or less than daily consumption, but the findings were statistically not significant.

Data between fish intake and SGA occurrence have found to be inconsistent. A study done by Rogers et al^32^ found that women eating no fish had higher SGA rates than women with the most upper quartile of fish intake (AOR 1.85, 95% CI 1.44, 2.38). In contrast, an American study reported babies born to women with the highest quartile of fish intake with a lower mean birth weight.^33^ A recent French study^34^ said that fish intake before pregnancy was associated with increased birth weight only in women with high BMI. In contrast, another French study^35^ reported that women eating two or more fish meals weekly had reduced SGA while those eating two or more shellfish meals weekly had increased SGA. In the present study, we found that monthly or less intake of fish was associated with a higher risk of SGA occurrence than those who consumed fish at least weekly.

### 4.3 Maternal illness

Various maternal medical illnesses have been associated with the occurrence of SGA. In the present study, we found that anemia, oligohydramnios, and gestational diabetes were independent risk factors for SGA. Macrosomia is typically associated with diabetes, whereas vasculopathy (retinopathy and/or nephropathy and/or preexisting hypertension) is associated with increased SGA. Haeri et al^36^ in a prospective study of 340 diabetic women, reported 10‐fold high chances of having SGA depending on the type of vasculopathy. In contrast, Teixeira et al^14^ could not find any statistical significance of gestational diabetes and pregestational diabetes with SGA occurrence. Howarth et al^37^ also reported that diabetic women with vascular disease had a higher risk of SGA (OR 6.0, 95% CI 1.5, 23) babies. Our study, too, supported 2‐fold increased SGA in diabetic mothers (OR 2.87, 95% CI 1.48, 5.55). The higher risk of SGA in diabetic mothers in our study may be due to vasculopathy in those mothers.

A recent systematic review did not find an overall increase risk of SGA babies with maternal diseases, but certain types, particularly cyanotic congenital heart disease had a higher risk.^38^ In our study, directionally, there was a higher risk of SGA among those with maternal cardiovascular disorders, which may be explained due to reduced placental blood flow in those women.

There was no statistical significance of maternal thyroid disease and SGA in the present study. Few earlier studies^39^, ^40^ have reported a positive association between hypothyroidism and low infant birth weight. The reason why previous studies might have shown the association could have been due to premature delivery as a confounding factor. Therefore, it is difficult to conclude the association between thyroid disease and SGA when most studies have only used low birth weight as the outcome. Hence, a greater understanding of SGA's risk factors is necessary, which may help reduce the prevalence of these conditions and the associated risk of infant mortality and morbidity.

## 5 STRENGTH AND LIMITATIONS

The study's strength was its large sample size and the inclusion of lifestyle, environmental risk factors, and various medical illnesses of the mothers. Despite the large sample size, this study had few limitations. The outcome of SGA babies could not be studied in this study, which would have helped us understand the disease's exact burden. The risk factors considered in this study were limited. We could not study many other risk factors that could act as confounders to the other risk factors investigated in this study. This study was a single center hospital‐based research and is visited from Nepal's hilly and plain regions and is unlikely to be representative of the entire population of Nepal; however, this region represents over 50% of the Nepalese people.

## 6 CONCLUSIONS

The prevalence of SGA in Nepal is high, with female‐SGA more common than the male‐SGA. Gestational diabetes (more likely females with vasculopathy), oligohydramnios, and maternal anemia were independent factors related to SGA babies' occurrence. Solid fuel use during cooking and smoking was also strongly associated with a higher risk of SGA babies. However, educated mothers and adequate sleep during pregnancy (10‐14 hours) were inversely related to SGA occurrence. Daily fruit consumption and daily high carbohydrate diet intake were protective factors in SGA occurrence. Information and education must be made available on these essential modifiable lifestyle factors.

## CONFLICT OF INTEREST

The authors declare that there is no conflict of interests.

## AUTHOR CONTRIBUTIONS

Conceptualization: Nagendra Chaudhary, Shree Narayan Yadav, Suresh Kumar Kalra, Binod Kumar Gupta, Sandeep Shrestha, Om Prakash Kurmi

Data Curation: Nagendra Chaudhary, Shree Narayan Yadav, Binod Kumar Gupta, Om Prakash Kurmi

Formal Analysis: Nagendra Chaudhary, Suresh Kumar Kalra, Santosh Pathak, Sandeep Shrestha, Matthew Patel, Imran Satia, Steven Sadhra, Charlotte Bolton, Om Prakash Kurmi

Investigation: Shree Narayan Yadav, Suresh Kumar Kalra, Binod Kumar Gupta, Sandeep Shrestha

Methodology: Nagendra Chaudhary, Shree Narayan Yadav, Suresh Kumar Kalra, Santosh Pathak, Binod Kumar Gupta, Sandeep Shrestha, Matthew Patel, Om Prakash Kurmi

Project Administration: Suresh Kumar Kalra, Sandeep Shrestha

Resources: Shree Narayan Yadav, Suresh Kumar Kalra, Binod Kumar Gupta, Steven Sadhra, Om Prakash Kurmi

Software: Nagendra Chaudhary, Shree Narayan Yadav, Matthew Patel, Imran Satia, Steven Sadhra, Charlotte Bolton, Om Prakash Kurmi

Supervision: Nagendra Chaudhary, Suresh Kumar Kalra, Binod Kumar Gupta, Sandeep Shrestha, Matthew Patel, Imran Satia, Steven Sadhra, Charlotte Bolton, Om Prakash Kurmi

Validation: Nagendra Chaudhary, Suresh Kumar Kalra, Santosh Pathak, Binod Kumar Gupta, Matthew Patel, Steven Sadhra, Om Prakash Kurmi

Visualization: Nagendra Chaudhary, Shree Narayan Yadav, Santosh Pathak, Binod Kumar Gupta, Imran Satia, Charlotte Bolton, Om Prakash Kurmi

Writing—Original Draft Preparation: Nagendra Chaudhary, Shree Narayan Yadav, Suresh Kumar Kalra, Santosh Pathak, Binod Kumar Gupta, Sandeep Shrestha, Matthew Patel, Imran Satia, Steven Sadhra, Charlotte Bolton, Om Prakash Kurmi

Writing—Review & Editing: Nagendra Chaudhary, Shree Narayan Yadav, Suresh Kumar Kalra, Santosh Pathak, Binod Kumar Gupta, Sandeep Shrestha, Matthew Patel, Imran Satia, Steven Sadhra, Charlotte Bolton, Om Prakash Kurmi

 The corresponding author, Dr. Nagendra Chaudhary, had full access to all of the data in this study and takes complete responsibility for the integrity of the data and the accuracy of the data analysis.

## TRANSPARENCY STATEMENT

The corresponding author, Nagendra Chaudhary, affirms that this manuscript is an honest, accurate, and transparent account of the study being reported; that no important aspects of the study have been omitted; and that any discrepancies from the study as planned (and, if relevant, registered) have been explained.

## Data Availability

The data that support the findings of this study are available from the corresponding author upon reasonable request.
